# IoT terminal security assessment system based on improved assessment method

**DOI:** 10.1371/journal.pone.0256881

**Published:** 2021-09-08

**Authors:** Zhe Liu, Zehao Jin, Chenhan Shangguan

**Affiliations:** School of Cyber Science and Engineering,Wuhan University, Wuhan, Hubei, China; University of Pisa, ITALY

## Abstract

The Internet of Things (IoT) technology is widely used and has been improved in research. However, due to the extensiveness of IoT technology, the heterogeneity and diversity of the device structure, the number of attacks against IoT has increased dramatically, so we need a method that can effectively and actively determine safety. Considering the diversity of the terminal structure of IoT, a security method for the IoT terminal based on structural balance, method objectivity, and reliability is currently a challenging task. This paper introduces the idea of rate of change in mathematics into trust analysis, and forms three attribute sets based on trust interval and rate of change: discrete interval, change range, and change frequency. By calculating the above attributes of the entity’s trust value, the entity’s trust situation is obtained, and an overall assessment of the terminal entity’s trust situation is made from the three levels of completeness, accuracy and objectivity. Under the premise of reducing encryption and other means, the above method can evaluate the trust state of the IoT terminal from the perspective of the data, and this evaluation method can provide a basis for the judgment of the IoT terminal more objectively and accurately.

## 1. Introduction

### 1.1. IoT needs security

The concept of the IoT was formally proposed in the 1990s [1–3]. When entering the 21st century, the goal of the IoT is to enable objects in the physical world to communicate with the digital or network world [4]. Up to now, the vision of the IoT is based on research in many fields, including big data, machine learning, edge computing, wireless sensor networks, etc.

However, while realizing the above-mentioned vision, the IoT is also facing increasingly serious security problems. In 2016, US FBI information security experts found that wireless embedded medical devices such as pacemakers and insulin pumps on the market currently have exploitable security vulnerabilities [5]. In the same year, the botnet launched a DDOS attack on the American domain name resolution service provider Dyn by controlling a large number of IoT devices, which caused a large area of network disconnection in the eastern United States, and many popular websites stopped serving [6]. In agricultural and industrial environments, if the IoT sensors are operating normally, they may not be inspected for a long time, and they are likely to be directly captured by attackers [7]. In recent years, there have also been many attacks in the IoT field, ranging from targeting individual user attacks (such as by using video baby monitors in smart homes) to nationwide attacks (such as attacks triggered by IoT botnets) [8–11].

As the IoT needs to face a large number of attack threats, IoT security has become an independent and important research direction. Shantanu Pal et al. [12] divided the security risks of IoT. They believe that many expectations of scalability, availability, connectivity, and flexibility can be realized in a way that can be practical and comprehensive, and IoT security system can be realized on this basis. Chen IR et al. [13] proposed to evaluate the credibility of other devices in the mobile device’s network to dynamically change the security configuration of the mobile device, thereby reducing its risk of being attacked. Llie-Zudor E et al. [14] proposed that mobile devices can use their location information to complete self-authentication. The authentication requires operations that can be performed only when the device is in a specific area. The program can be simplified by self-authorization based on the device’s location information in the certification process. SAJJAD SM et al. [15] and others pointed out that there are security vulnerabilities in the transmission link layer of IoT. Attackers can launch synchronization attacks, tampering attacks, and energy exhaustion attacks against these vulnerabilities.

The classic IoT security still relies mainly on security reinforcement methods such as terminal device entity verification, network transmission encryption protection, and data encryption storage. However, due to the massive heterogeneity of IoT, it is necessary to make some adjustments to terminal security based on the nature of IoT.

The concept of trust is an important part of computer security [16]. Since the entities involved in the interaction of the IoT may be unrelated services and equipment, trust evaluation becomes particularly important in reference [5, 7, 17–20]. At the same time, the determination of the identity of the interacting party based on trust evaluation and the security and practicality based on the concept of trust have also become two independent research directions. Trust management strives to identify reliable nodes, considers the risks brought by these nodes to be acceptable, and limits the interaction relationship to only those nodes that include such acceptable risks. With the aid of big data and other tools, the concept of trust can effectively reduce the hardware requirements in the field of IoT terminal entities, and at the same time can reduce other hardware overheads caused by the implementation of trusted computing.

### 1.2. Related work in IoT trust analysis

This section reviews trust research and the exploration of the introduction of trust in IoT, and show that trust research based on IoT has further research significance.

Early trust models are mostly binary trust with probability distribution [21] and multi-level trust without probability distribution [22]. The typical representative of binary trust is the two-level discrete-level model [23], which records the trustworthy state as "1" and the unreliable state as "0"; multi-level trust refers to the trust state divided into multiple levels, the typical representative of which is the multi-level discrete level model [24]. The binary discrete trust level model is easy to evaluate and easy to accumulate, but the multi-level discrete trust model can express the trust distribution state more effectively.

In further research, scholars introduced the probability distribution model to trust analysis, taking into account the uncertainty of the trust process and made a great improvement in trust evaluation. L. Xing [25] proposed a method based on a binary decision diagram to evaluate the trust between two parties, but it is only applicable to the binary trust model and cannot make an accurate judgment on the overall trend of trust. Sherchan [26] proposed a tidal trust algorithm, which collects trust data from recommended paths, and used a weighted average method to calculate the overall trust value, and infers the trust relationship through continuous levels. However, this type of method is used in complex networks or designated paths, and will be certain restrictions on trust evaluation. D. Chen et al. [27] proposed a trust management model for the IoT based on fuzzy reputation, but the use environment of this model is relatively single, and can only evaluate the objective attributes of packet forwarding ratio and energy consumption. Jiang et al. [28] proposed a flow-based trust evaluation method, which uses network flows to resolve path dependence and uses flow leakage associated with each node to model trust decay or propagation. Boa and Chen proposed a dynamic trust management system and extended it to the service composition based on trust in IoT papers [29, 30]. The model used a weighting factor to determine the relative importance of the recommendation based on the trust level of the node that provides the recommendation, so as to improve the flexibility of the system relative to the ratio of good peers and malicious peers.

In the field of trust evaluation of the trust status of end entities, Anuoluwapo A. Adewuyi [31] et al. proposed a dynamic trust model called CTRUST. The model used trust criteria as the basis for evaluation. The evaluation of the current and past direct interactions between entities, as well as the advisory evaluation of other nodes, calculated the trust value, which was used to evaluate and manage the trust between entities in collaborative applications in IoT. Rinki Rani et al. [32] proposed an energy-efficient trust evaluation (EETE) scheme, which used a hierarchical trust evaluation model to mitigate the malicious influence of illegal sensor nodes and limit the spread of trust requests within the network to reduce cluster sensors Energy Consumption. Kamran Ahmad Awan et al. [33] used trust parameters to identify malicious and infected nodes in the field of agricultural IoT. The trust mechanism based on trust parameters is based on the event mechanism and predefined time, and the absolute trust is determined and maintained based on the past trust. Liang Zhao et al. [34] builds a distributed analysis framework based on the fog-based IoT system, uses all the computing power of all interactive parties, and uses a privacy protection protocol based on a cryptographic scheme to protect privacy. Zhiquan Liu et al. [35] proposed a trust cascade-based emergency information distribution (TCEMD) model in the field of in-vehicle IoT, which integrated entity trust value into information evaluation in a more effective way. This method used trust certificates and trust evaluations as the basis for the weight of trust values, and analyzed the robustness of the TCEMD model against multiple attacks and malicious behaviors, fault tolerance, compatibility with several special situations, and theoretical analysis of incentive mechanisms.

Overall, most of the existing trust models in IoT are mainly based on recommendations and have the inherent risks highlighted above. In further research, trust is necessary to parameterize and evaluate quantitatively in a mathematical way.

### 1.3. The work and objective of the paper

As we know from the analysis above, a well-defined trust model for IoT applications is required. The trust score is a performance index based on functional attributes related to the collaboration context. At the same time, the mathematical change idea and frequency concept are used to analyze the trust value, and further through the trust analysis object possible security status of networked entities. In the common trust evaluation field of IoT, this article makes adjustments to IoT trust research based on the elements of trust value, so that the IoT terminal security evaluation can adapt to a broader platform, and can more objectively build IoT based on the concept of trust terminal security system. The main work and contributions are as follows:

- Reduce the hardware impact of IoT terminal entities. Since this article does not involve specific hardware structures or security modules in trusted computing, there is no need to set the root of trust in advance. This move can reduce the overhead from a hardware perspective, allowing terminal entities to more efficiently concentrate on interactive processes such as data collection and transmission.
- Use the attributes of continuous trust values and discrete evaluation sets to effectively evaluate the trust status. This paper introduces the concept of continuous trust value, and builds a multi-level discrete level model based on the formation of trust change rate; integrates the multi-level discrete trust model and the multi-level trust model of continuous probability distribution in the security assessment of the terminal entity of the IoT to trust the entity. The situation is measured in real-time to obtain the trust change of the trust entity in real-time, and the trust situation of the terminal entity in the IoT environment is modeled.
- Reduce the contingency brought by the trust value. Considering that the sudden change of trust value and other factors have an impact on the accuracy of trust-based security evaluation, this paper introduces the concept of rate of change to evaluate the trust state from the perspectives of the magnitude and frequency of change; while improving the accuracy of trust evaluation. Then avoid the accuracy impact caused by accidental factors such as trust mutation.
- Expand the safety evaluation system based on the concept of trust. The trust value dimension is extended to multiple dimensions of trust interval, trust change amplitude, and trust change frequency, and from the above-mentioned multiple dimensions, the possible security status of the terminal entity is evaluated together, which further expands the security evaluation of the trust concept.
- Realize the adaptation of the evaluation method through big data. Due to the heterogeneity and massiveness of the hardware environment of IoT, and the existing evaluation methods have more diversity, such as direct cross-platform transplantation and application involves multiple impacts on the hardware environment and parameter weights. Research on trust evaluation from mathematical attributes, reduces the dependence on weights and hardware conditions to make improvements in objectivity and adaptability.

The overall structure of the subsequent parts of this article is as follows: The second part introduces the general idea and concepts. The third part analyzes and derives the trust and trust changes involved in this article. The fourth part is the result analysis. The fifth part summarizes and analyses the future direction of work.

## 2. Materials and methods

### 2.1. Overall

This paper makes research in the field of IoT terminal security. The proposed method starts from the full cycle of the trust measurement evaluation of the terminal entity, and minimizes the hardware overhead of the IoT terminal entity. Then on the premise of determining the metric standard, we build a discrete interval evaluation set, trust change range, and trust change frequency, and use this as the set of attributes for trust analysis, real-time measurement, and calculation of the trust value, and classify the trust value into the corresponding evaluation set. Then evaluate the entity’s possible trust based on the frequency of the change magnitude together with the change rate and the trust discrete interval reliability status.

This paper will implement the trust evaluation based on the rate of change through the following work:

- Construct appropriate trust metrics. Based on previous work, we use a formed metric as the trust metric [31]. And build a trust value calculation model, using this as a standard to calculate the trust value in real-time.
- Calculate the rate of change of trust in a given time frame. We introduce the univariable of time, obtaining the instantaneous rate of change of the trust degree at that moment in a small interval, and compare the obtained rate of change, then compare the entities with larger fluctuations and frequent jitters. We make key monitoring and determine the possible security status of the terminal entity at that moment in conjunction with the evaluation of the multi-dimensional trust discrete interval constructed subsequently.
- Construct a multi-dimensional trust discrete interval evaluation set. To reduce the difficulty of evaluation and form a more effective evaluation of trust value, this paper uses the idea of discrete evaluation to divide the trust value into several trust discrete interval evaluation sets, and the trust value obtained by the real-time measurement is passed through the cluster analysis algorithm with realizing the classification of evaluation sets.
- Form a mechanism for determining the extent of change. After forming the discrete interval evaluation set, we make a preliminary analysis of the rate of change of the trust value. Based on the analysis result, we determine the criterion of the magnitude of the change through the floating range of the evaluation set.
- In a given time range, record the frequency of large changes and fluctuations in the trust value. Based on the change range determination mechanism, the frequency of the substantial increase or decrease of trust is calculated in a given time, and the proportion of the total and the increase or decrease of trust is calculated to determine the trust state that the entity may be in.

**Fig 1:**
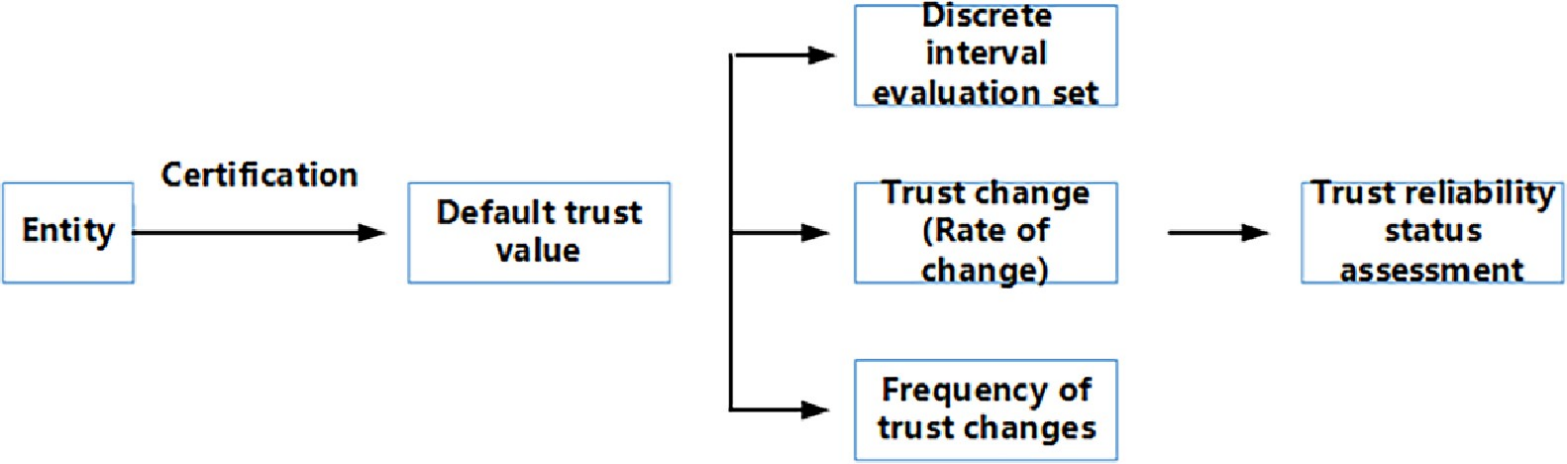
Flow chart for evaluating the trend of trust changes.

According to the stated goals, the evaluation flowchart designed in this paper based on the trend of trust changes is as Fig 1.

### 2.2. Concepts related to change analysis

According to the overall idea in 2.1, this section defines the default rules and related concepts involved in the trust change assessment.

The rules are defined as follows:

The trust value is continuous in the interval [0,1]. According to the view of trust value in the previous literature, we adopt continuous trust value to measure trust relationship. Take 0 as completely untrustworthy, 1 as completely trustworthy, and float within the range of [0,1].

The trust element is a positive element system. The trust evaluation model used in this article is derived from social networks. So the trust elements and trust value are measured in the same way, using a positive evaluation system. The trust elements of the positive evaluation system can be consistent with the meaning of trust value measurement.

The initial state of the authenticated trust has a default value of 0.5. According to the analysis and description of the trust value in the previous literature, to have a clear concept of raising and lowering the trust analysis, we select the intermediate value in the trust evaluation range as the basic trust value after the zero-trust network default authentication.

The conceptual part is defined as follows:

**Entities**: Refer to the mathematical definitions of entities on other platforms. The entities in this article specifically refer to terminal entities in IoT field. When performing trust evaluation on entities, the above entities are users who use the evaluation function, so we can refer to definitions in other fields and use User as the representation of the entity.

**User trust**: The user trust in this article is based on the calculation of various indicators of data interaction after the terminal entity completes the authentication. This article uses Trust (T) to represent user trust.

**Trust measurement characteristic benchmark [30]**: The entity trust value T represents the trust degree of the entity user in the overall environment of IoT. According to the entity trust, the requirements of the elements and the comprehensive definition of trust characteristics. This article uses the characteristics mentioned by the previous standard as the benchmark of trust measurement. The specific content is described in the third part.

**Trust change model**: A comprehensive mathematical model based on probability theory, Boolean algebra, and trust measurement, used to measure trust changes. In this model, the trust level of the terminal entity is determined by the initial trust situation.

Since the trust degree selected in this paper is a continuous function in the range of [0,1], which is comprehensively judged by the trust degree fed back by the real-time measurement, the trust distribution in this topic is a continuous value instead of the discrete trust evaluation work before value.

**Initial trust value**: The initial trust value is the same as the default trust value, and the initial trust value is 0.5 which is based on the normal distribution and other mathematical models.

**Trust discrete interval evaluation set**: Within a given trust interval, according to changes in trust, the level of trust value is differentiated. By referring to the current existing literature on the distribution of trust levels, this paper divides the discrete intervals of trust into complete trust, high trust, default trust, relatively untrustworthy, highly untrustworthy, and completely untrustworthy from high to low.

**Trust change rate**: In the time range of given two points t_0_ and t, the ratio of the difference between the trust degree T and T_0_ to the time change is defined as the trust change rate T´; it represents the degree of trust change in a certain period time seriousness.

**Trust fluctuation threshold**: The interval range of the discrete interval evaluation set within the time range of given two points t_0_ and t, expressed by λ.

**Trust fluctuation frequency**: The sum of the frequencies exceeding the fluctuation threshold within the given time range of two points t_0_ and t, expressed as f; if the trust fluctuation changes in a discrete interval, the fluctuation frequency is also calculated.

## 3. Research on trust calculation method

Based on the overall ideas and definitions of the second part, this part derives and describes the calculation methods of trust value calculation, trust change determination, discrete interval evaluation set and fluctuation.

Before introducing the definition of calculation in this chapter, we will first explain the symbols and definitions used in it, as shown in Table 1.

**Table 1:** Symbol definition table used in this paper.

Symbol	Definition
i	Starting point of trust value calculation time
j	End of trust value calculation time
C	The trust measure is between i and j at a certain time point
P(p)	A collection of parameters and definitions of trust attributes
x	Sort values for trust elements
W	The importance of p as determined by i
V	The trust score of j, as determined by i, on parameter p at time C
F	Weighted sum function
T^,^	The change rate of trust value in a certain period of time

### 3.1. Obtaining trust value

In this article, we take the cumulative average method [31] of each element in the third part of the trust measurement characteristic benchmark to achieve the calculation of the trust value. According to the above principles, the calculation definition of the trust value is as follows:

$$T_{i}\equiv [P,W_{i},V_{i}]\;\forall i\in C$$ (1)

In the definition of trust, the meaning of each part is as follows:

Ti: The score of trust summary at time i, it could be calculated as shown in formula 2.


$$T_{i}=\sum_{x=1}^{n}\omega_{i}(p_{x})\times V_{i}(p_{x})\;\forall i\in C,p_{x}\in P$$ (2)

P: A collection of parameters and definitions of trust attributes. We take the attribute aggregation represented by the literature [31]. So in this paper, P is used as an intermediate parameter to participate in the aggregation of trust value formation, but does not directly participate in the trust analysis.

Wi: The importance of p as determined by i,

$$W_{i}=\{w_{i}(P_{1}),\;w_{i}(P_{2}),\;w_{i}(P_{3}),\;\ldots ,\;w_{i}(P_{n})\}$$ (3)

Vi: The trust score of i on parameter p.at time C.


$$V_{i}=\{V_{i}(P_{1}),\;V_{i}(P_{2}),\;V_{i}(P_{3}),\;\ldots ,\;V_{i}(P_{n})\}$$ (4)

The specific calculation methods refer to the specific implementation steps in [30], and use this as the benchmark for obtaining the trust value.

After determining the definition of trust degree, the trust measurement and discrete interval classification are realized through the following steps, and the trust discrete interval evaluation set is constructed:

Step 1: Based on the concept of zero-trust network, the terminal entity is authenticated when it is started. The terminal entity that has passed the authentication can be considered to have completed the initial authentication work and assigned a default trust value. In the setting of this article, the default trust value is based on the principle of mathematical normal distribution, and the value is 0.5, which is the default trust degree located in the middle line.
Step 2: Record the data interaction and the behavior of the terminal entity, and perform measurement analysis according to the behavior situation at the same time to form a trust change table in the behavior process.

Based on the definition of the normal distribution interval in mathematics, this article defines the completely reliable value as 1, and the completely unreliable value as 0. At the same time, the highly reliable value is [0.75,1), the more reliable value is (0.5,0.75], and the less reliable value is [0.25, 0.5), and the high unreliability is (0, 0.25).

### 3.2. Calculation of trust change rate

In this article, the calculation method of trust change rate based on trust value is as follows:

Given a terminal entity, its trust degree is represented by T. For other terminal entities, it also has its trust degree. Under the above conditions, the overall trust value of the entity in the network environment depends on the algebraic synthesis of the trust value of other interactive nodes.

We use T to represent the trust value of the terminal entity, and believe that the trust value changes with time t, to construct the definition, and *T*^,^ represents the rate of change to measure the trust change.

According to the previous definition of trust value, the degree of trust between 0 and 1 adopts a continuous probability distribution to ensure the continuity of the trust value measurement. In this distribution model, it can be understood within a given small interval *T*^,^ for instantaneous discrete.

In a trust-based IoT environment, the trust level defined in this article is reflected by real-time trust value measurement. The trust value generated by real-time measurement can reflect the overall trust situation of the terminal entity in the IoT environment, and it can also reflect the trust situation of the interaction between entities. If the trust value is used as a reflection of the interaction, it can be based on direct trust value or indirect trust value.

Select the trust change interval to be analyzed, first determine the trust starting point i and the trust timestamp ending point j, and determine the initial trust value T_0_ and the end trust value T in the interval according to the time range.

According to the definition of the trust change rate, the trust change rate T^,^ is obtained after selecting the trust change interval to be analyzed, which represents the change of the trust degree of the entity T during the period time from i to j.

If it is required to obtain the change T of the trust degree of the entity T at a certain time, it can be realized by using the derivative definition. At this time, the obtained change rate T^,^ represents the instantaneous trust change at time t.

In the analysis of trust change, there are also two types of change rate, namely point change rate, and period time change rate. The point change rate reflects the trust change at time i, and the period time change rate reflects the overall trust change in a certain period of time.

The point change rate and period time change rate are obtained by the following formula:

$$T^{,}=\frac{dTrust[T_{ij},P,W_{ij},V_{ij},F]}{dC},\forall i,j\in C$$ (5)

We use Birnbaum’s measure in [36] is the most classic one and has been extended for multi-state components. The formulas (5)(6) illustrates the Birnbaum measure of a k-state component i, denoted by Ti.


$$T^{,}=\frac{[T_{ij},P,W_{i},V_{ij},F]-{\left[T_{ij},P,W_{i},V_{ij},F\right]}_{0}}{j-i}$$ (6)

The above formula uses Birnbaum’s method and integrates mathematical properties to evaluate the entity’s trust value. Under the condition that the two parameters i and j are fixed, the above formula represents the comprehensive state of the trust value at time t between the two orders of magnitude i and j; and (5) and (6) respectively start from point time and interval time. Define the change of the trust value, which represents the point change and interval change at a certain moment within the i and j parameters.

Under the conditions of specific needs, changes in the selected trust levels will also be different. In this article, the overall trend uses the time period change rate, but the point change rate is also used for precise observation.

Introducing the rate of change into the trust discrete interval evaluation set, then the expansion results of the one-dimensional evaluation set in Table 2 are shown in Table 3.

**Table 2:** Trust classification of trusted entities.

Confidence	Complete	Highly	Relatively
Interval	Reliable	Reliable	Reliable
Default	Less	Highly	Complete
Reliability/trust	Reliable	Unreliable	Unreliable

**Table 3:** Trust evaluation form after introducing rate of change.

Trust Change Character- istics Confidence Interval	No change	Small changes up and down	Stable linear rise	Linear decline and stability	Rate of change jitter
**Complete Reliable**	Complete Reliable	-	-	-	-
**Highly Reliable**	Highly reliable	Highly reliable	Highly reliable	Further Evaluation	Further Evaluation
**Relatively Reliable**	Relatively Reliable	Relatively Reliable	Relatively Reliable	Further Evaluation	Further Evaluation
**Default Reliability/Trust**	Default Reliability	Default Reliability	Further Evaluation	Unstable State	Further Evaluation
**Less Reliable**	Less Reliable	Less Reliable	Further Evaluation	Unstable State	Unstable State
**Highly Unreliable**	Default Reliability	Default Reliability	Further Evaluation	Unstable State	Unstable State
**Complete Unreliable**	No Trustworthy	-	-	No Trustworthy	-

### 3.3. Fluctuations in trust changes

In this section, we design the following algorithm for the fluctuation frequency of trust changes to realize the statistics of the entity’s trust fluctuations within a given-time frame.

When we construct the discrete interval evaluation set, based on the normal distribution and predecessors’ understanding of the evaluation set, we set the range of each trust evaluation interval to 0.25. When actually making a trust evaluation, we also need to be vigilant about trust entities that have not exceeded the evaluation set but have experienced a jump in trust. Considering the stratification in the same interval, we use 0.10 as the trust change threshold within a unit time. If this threshold is exceeded within a unit time, we will mark it and conduct further analysis.

Algorithm1 Preliminary classification of trust fluctuations

 (this algorithm initially divides trust fluctuations into fluctuations within the threshold range and fluctuations beyond the threshold)

The trust value calculated by the algorithm of the preamble 3.3 is the input value of this algorithm

The output is the classification of trust value and fluctuation

If (trust change rate dt>=0.10) then

Record the moment and the rate of trust change in the range of the trust fluctuation set

Else if (across a confidence interval) then

Record the moment and the rate of trust change in the range of the trust fluctuation set

Else

 It is not considered as an excessive fluctuation range of trust

and the reliability of trust is evaluated through the discrete interval of trust

According to the algorithms mentioned in this part, we initially divide trust changes into small fluctuations within a limited discrete interval, and other forms of fluctuations that require further classification. Other forms of fluctuations need to record the frequency of fluctuations, but the above-mentioned fluctuations need to be further classified, which is what algorithms 2 and 3 do.

Algorithm 2 further categorizes the evaluation set transformation of trust change, thereby determining the approximate interval of the trust value transformation, and recording the fluctuation frequency of the interval change; in the above classification, we pay more attention to the previous types of trust According to the general cognitive requirements of the security concept, the trust state before reaching the default trust is calculated as unreliable.

The Algorithm 3 is to make further statistics on the frequency of the change rate exceeding the threshold value and record the frequency of the threshold value change.

Algorithm2 Volatility classification beyond the evaluation set

 (this algorithm distinguishes small rate of change, but volatility across two evaluation sets)

The algorithm in the preamble 1 calculates the volatility set as the input of this algorithm.

The output of the algorithm is the frequency of small fluctuations across the two evaluation sets.

If (trust change rate dt>=0.10) then

Record this moment and the rate of trust change in the set of trust crossing threshold fluctuations

Else if (dt>0) then

Case 0: High trust becomes full trust;

Case 1: Relative trust becomes high trust or complete trust;

Case 2: The default trust becomes more trust;

Case 3: Less trustworthy becomes default trust or more trust;

Case 4: Highly untrustworthy becomes more untrustworthy;

Case 5: Totally untrustworthy becomes highly untrustworthy;

Else

Case 0: Full trust becomes high trust;

Case 1: High trust or complete trust becomes more trust;

Case 2: The more trust becomes the default trust;

Case 3: More trust or default trust becomes less trustworthy;

Case 4: Less trustworthy becomes highly untrustworthy;

Case 5: Highly untrustworthy becomes completely untrustworthy;

and the reliability of trust is evaluated through the discrete interval of trust

Algorithm3 Volatility classification beyond the threshold

If (trust change rate dt>=0.10) then

Record the frequency of trust increase, and record the discrete interval

Else

Record trust reduction frequency, and record discrete intervals

Do accumulate trust increase frequency and decrease frequency, and record the initial trust value and the end trust value

and the reliability of trust is evaluated through the discrete interval of trust

Based on the above algorithm and accumulating the frequencies in 2 and 3, the comprehensive trust interval evaluation set can evaluate the general state of trust reliability of the trusted entity, and take corresponding measures based on this state.

## 4. Experiment and evaluation

In this part, we analyze the trust of the terminal entity based on the design ideas mentioned above. In the simulation evaluation, the previous data set [31] is used, and the results are analyzed through the trust formation algorithm.

Specifically, in the trust evaluation of this article, we first determine a trusted entity node to be evaluated. Then the trust status of the node is recorded in real time, and the interval evaluation set where the node is located is marked. Then within a given time range, the trust change rate of the point is obtained in the form of the point change rate, and compared with the threshold value to record whether the threshold value is exceeded. If it does not exceed the threshold value but crosses two evaluation sets, it is also regarded as exceeding the threshold processing. Finally, the frequency of exceeding the threshold in a given time is summarized, and based on the rate of change and the frequency of exceeding the threshold, the safety and reliability state of the terminal node may be judged together.

Considering that the focus of this article is to evaluate the security and reliability of the terminal entity, the follow-up operations to obtain the evaluation results and the components of the pre-order trust value are not the focus of this article.

### 4.1. Obtaining trust value

As for the calculation of trust value, this paper deduces the calculation formula of trust value in Section 3.1, and makes dynamic modeling based on this model. In this experiment and analysis, we measure each entity with different trust states in real time, and generate trust values based on the measurement results.

In addition, we retain the evaluation set of discrete trust interval in trust evaluation, and determine the discrete trust interval of measurement trust value through the evaluation definition of discrete trust interval in 3.1, which is used as the basis for the preliminary evaluation of trust value [37].

For the convenience of analysis, the sampling analysis method is adopted in this paper, and the time stamp of application analysis is selected as 20 seconds, which is convenient for statistical analysis. At the same time, it should be noted that this part of the analysis is based on the measurement results. The actual trust value obtained and measured by the trust value is much larger than the centralized basic state described later.

### 4.2. Analysis of change rate

**Fig 2:**
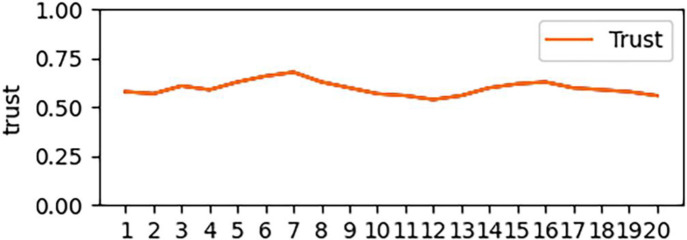
Stable entity trust change.

According to the trust value obtained by real-time calculation and measurement in Fig 2, we construct the following trust change states and analyze them separately.

Fig 2 and Table 4 show the state of trust change under the premise of stable change range. As can be seen from Fig 2, the overall reliability of the trust entity shown is **Relatively Reliable**, and the maximum change of trust at a certain time is only 0.05. At this time, because the change of trust is not obvious, the trust value and the change of trust are basically the same.

**Table 4:** Stable entity trust changes in Fig 2A.

Time	1	2	3	4	5	6	7	8	9	10
Trust(T)	0.58	0.57	0.61	0.59	0.63	0.66	0.68	0.63	0.60	0.57
Trust Change (T^,^)	-	-0.01	0.04	-0.02	0.04	0.03	0.02	-0.05	-0.03	-0.03
Time	11	12	13	14	15	16	17	18	19	20
Trust(T)	0.56	0.54	0.56	0.60	0.62	0.63	0.60	0.59	0.58	0.56
Trust Change (T^,^)	-0.01	0.02	0.02	0.04	0.02	0.01	-0.03	-0.01	-0.01	-0.02

**Fig 3:**
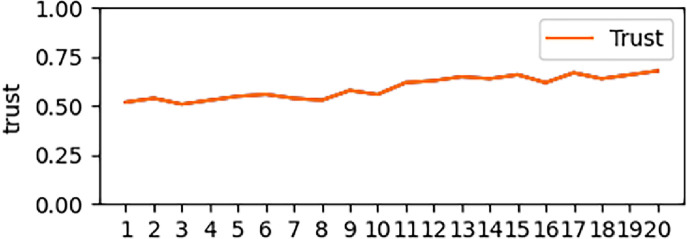
Steadily improvement of trust entities.

In Fig 3, the former is **General Trust**, and the latter is **Relatively Reliable**; but the rate of change is going to a stable state after a steady increase. Based on the change trend of trust and the degree of trust, the above picture can also be evaluated as a stable trust state.

The trust states shown in Fig 2 and Fig 3 are two ideal states in this study: robust and reliable trust. Without excessive trust fluctuations, if the entity appears in the above state, the trust state can be calculated directly according to the trust value.

**Fig 4:**
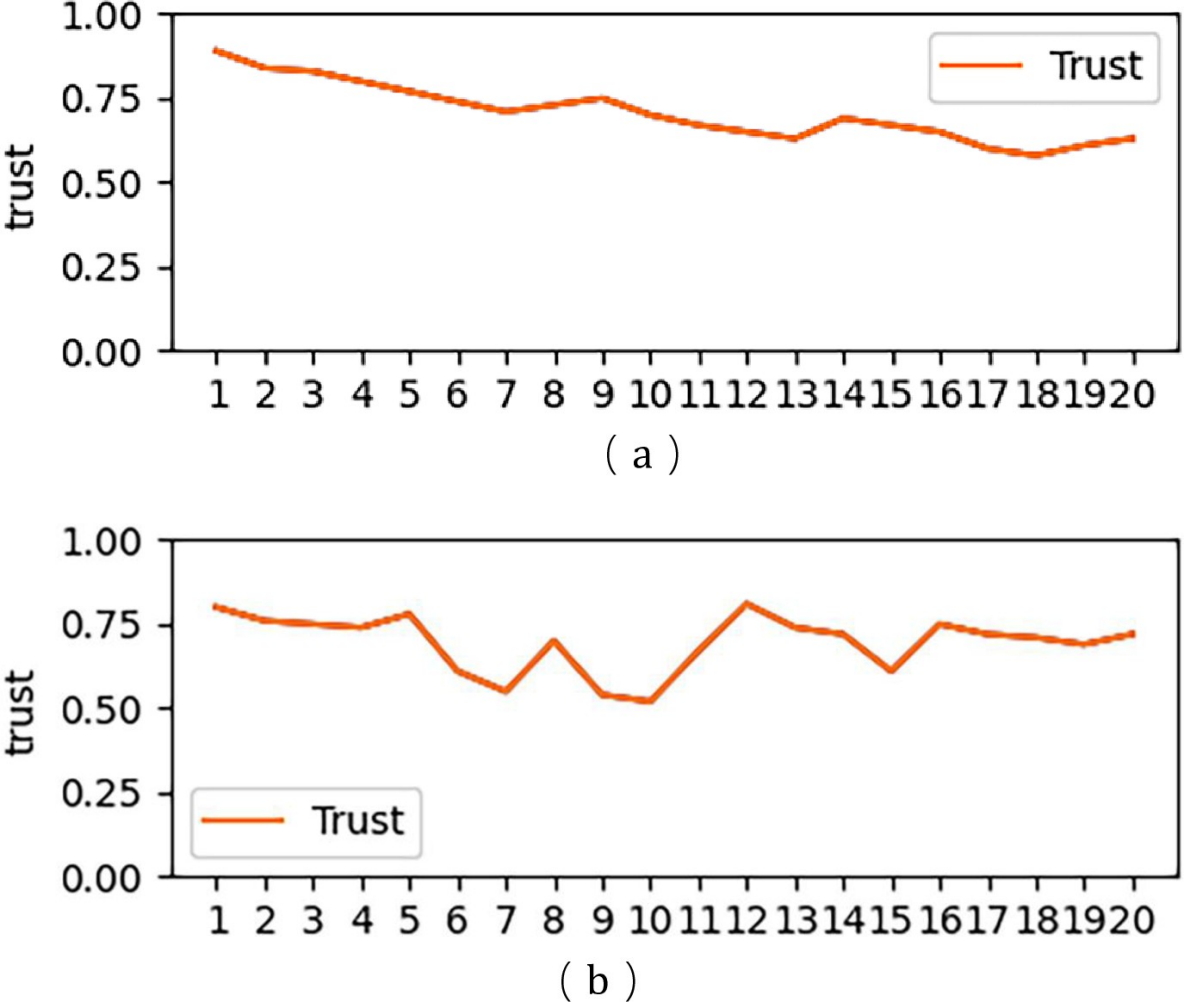
Changes in trust entities. (a: general trust; b: mutation trust).

However, the trust status in Fig 4 does not necessarily indicate that the end entity must be robust and reliable.

Fig 4 shows the trust changes of two entities that have experienced a decline in their trust status. If judged according to the trust values shown in the two pictures, the trust values of the above two entities are in default trust and high trust states, respectively. According to the conventional trust analysis method, it can basically be considered that the evaluation has been completed by doing this.

We made the trust value at each moment in Fig 4 into Table 5, and attached the trust change at each moment in Table 5. For the trust entity shown in Fig 4(A), the trend of change in this given period of time is a steady decline. In the process of decline, there will be an increase in some moments (for example, in 9 and 10 second from Table 5A), but the trust value and trust interval at the rising moment cannot be used as the entity trust evaluation benchmark. After we introduce the idea of change, we can conclude that the entity’s trust status has dropped significantly through the magnitude and frequency of change. Although there is an increase in a certain period of time, the frequency and amplitude of the increase are far less than the frequency and amplitude of the decrease. Therefore, for Fig 4(A), after the above analysis, although its trust value is still credible by default, it is still in a downward trend, so further evaluation measures need to be taken for this type of entity.

**Table 5:** 

A. Trust Entities in Fig 4A										
Time	1	2	3	4	5	6	7	8	9	10
Trust(T)	0.89	0.84	0.83	0.80	0.77	0.74	0.71	0.73	0.75	0.70
Confidence Interval	Highly reliable	Highly reliable	Highly reliable	Highly reliable	Highly reliable	**Further Evaluation**	Relatively Reliable	Relatively Reliable	Relatively Reliable	Relatively Reliable
Trust Change (T^,^)	-	-0.05	-0.01	-0.03	-0.03	-0.03	-0.03	0.02	0.02	-0.05
Time	11	12	13	14	15	16	17	18	19	20
Trust(T)	0.67	0.65	0.63	0.69	0.67	0.65	0.60	0.58	0.61	0.63
Confidence Interval	Relatively Reliable	Relatively Reliable	Relatively Reliable	Relatively Reliable	Relatively Reliable	Relatively Reliable	Relatively Reliable	Relatively Reliable	Relatively Reliable	Relatively Reliable
Trust Change (T^,^)	-0.03	-0.02	-0.02	0.06	-0.02	-0.02	-0.05	-0.02	0.03	0.02
B. Trust Entities in Fig 4B										
Trust(T)	0.80	0.76	0.75	0.74	0.78	0.61	0.55	0.70	0.54	0.52
Confidence Interval	Highly reliable	Highly reliable	Highly reliable	**Further Evaluation**	**Further Evaluation**	**Unstable State**	Relatively Reliable	**Unstable State**	**Unstable State**	Relatively Reliable
Trust Change (T^,^)	-	-0.04	-0.01	-0.01	0.04	-0.17	-0.06	0.15	-0.16	-0.02
Time	11	12	13	14	15	16	17	18	19	20
Trust(T)	0.67	0.81	0.74	0.72	0.61	0.75	0.72	0.71	0.69	0.72
Confidence Interval	**Unstable State**	**Unstable State**	**Further Evaluation**	Relatively Reliable	**Unstable State**	**Unstable State**	**Further Evaluation**	Relatively Reliable	Relatively Reliable	Relatively Reliable
Trust Change (T^,^)	0.15	0.14	-0.07	-0.02	-0.11	0.14	-0.03	-0.01	-0.02	0.03

For the entity shown in Fig 4(B), the trust value is also in the discrete interval of the default trust. But for this form, it can also be seen from the analysis of Table 6 that the trust change in Fig 4(B) has experienced large fluctuations with multiple frequencies and high frequencies. Although for this entity, the trust value of the initial form and the final form does not change much, but the trust change experienced by the entity in the middle has a large jitter. For example, the fluctuations in 7–8 seconds, 8–9 seconds and 15–16 seconds, even if they are in the discrete interval of default trust, but the large fluctuation of trust value is not a very stable security form. The corresponding evaluation has also changed from the default credibility of the original trust value to an unstable state of trust, and the evaluation of this type of entity will be more stringent than that in Fig 4(A).

**Table 6:** Stable entity trust changes in Fig 3A.

Time	1	2	3	4	5	6	7	8	9	10
Trust(T)	0.52	0.54	0.51	0.53	0.55	0.56	0.54	0.53	0.58	0.56
Trust Change (T^,^)	-	0.02	-0.03	0.02	0.02	0.01	-0.02	-0.01	0.05	-0.02
Time	11	12	13	14	15	16	17	18	19	20
Trust(T)	0.62	0.63	0.65	0.64	0.66	0.62	0.67	0.64	0.66	0.68
Trust Change (T^,^)	0.06	0.01	0.02	-0.01	0.02	-0.04	0.05	-0.03	0.02	0.02

**Fig 5:**
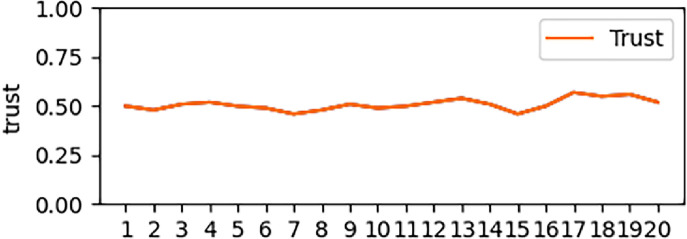
Changes in trust levels between default trust values.

The trust change in Fig 5 and Table 7 has a commonality with Fig 2 in that it tends to be stable, but the difference from Fig 2 is that the trust entity in Fig 3 has fallen outside the default trust value at some point. It is true that the trust value in this case is still a credible state, but the reason for falling out of the default trust value needs to be analyzed at an instant.

**Table 7:** Default trust values in Fig 5.

Time	1	2	3	4	5	6	7	8	9	10
Trust(T)	0.50	0.48	0.51	0.52	0.50	0.49	0.46	0.48	0.51	0.49
Confidence Interval	Default	Less	Further	Relatively	Relatively	Less	Less	Less	Further Evaluation	Further Evaluation
	Reliability	Reliable	Evaluation	Reliable	Reliable	Reliable	Reliable	Reliable		
Trust Change (T^,^)	-	-0.02	0.03	0.01	-0.02	-0.01	-0.03	0.02	0.03	-0.02
Time	11	12	13	14	15	16	17	18	19	20
Trust(T)	0.50	0.52	0.54	0.51	0.46	0.50	0.57	0.55	0.56	0.52
Confidence Interval	Further Evaluation	Relatively	Relatively	Relatively	Further Evaluation	Further Evaluation	Relatively	Relatively	Relatively	Relatively
		Reliable	Reliable	Reliable			Reliable	Reliable	Reliable	Reliable
Trust Change (T^,^)	0.01	0.02	0.02	-0.03	-0.05	0.04	0.07	-0.02	0.01	-0.04

For trust entities with mutations in trust, they can be considered credible from the perspective of trust value, but they need attention from the perspective of trust changes. However, the reliability of trust is not only determined by the magnitude of trust change, but also the frequency of change is one of the very important attributes. The frequency of change will be further elaborated in the next section.

### 4.3. Analysis of change frequency

We have made a brief analysis of several types of change modes based on comparison in 4.2. However, in the actual trust assessment, the performance of the change range is the synthesis of several basic states in 4.2. At this time, only the point change at a certain time can not represent the overall trend. Although we can measure the magnitude of change between two points to measure the real-time state of trust change, such analysis can not have a better estimation of the midway change.

We introduce the basic idea of frequency into the actual evaluation, and make some application in the basic state of the early stage. Then, the possible trust reliability state of the terminal entity in a given time range is analyzed by combining the previous discrete interval evaluation set and the change threshold.

**Fig 6:**
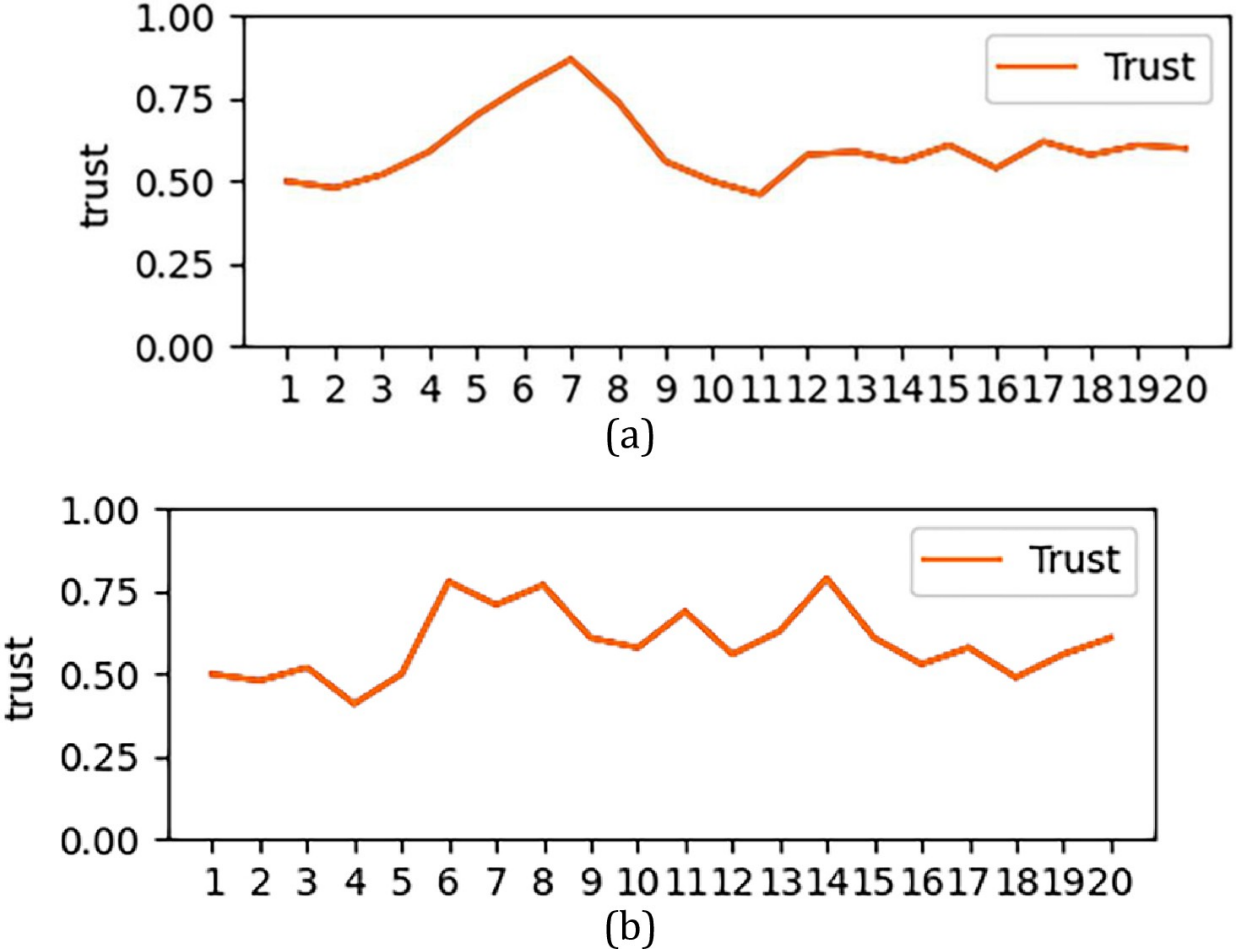
Comparison of trust status within a certain range.

Fig 6 is a state diagram of the change of trust in a certain period of time. In this state diagram of the change, we made a behavioral simulation of the entity that can affect a large change in trust, and performed a trust change at this time track and record in real time.

Fig 6(A) and 6(B) represent the trust change from the beginning of the default trust value to the end of 20 units, and Table 8 and Table 9 represent the change of trust amplitude and frequency. According to the current algorithm, the final trust value is 0.65, which is in a relatively trusting state. However, from the change rate and frequency of the two graphs, the trust state feedback in Fig 6(A) and Table 8A is relatively more stable. So in terms of the overall rate of change and frequency, the entity represented by a has a better credibility.

**Table 8:** 

A. Trust Entities in Fig 6A										
Time	1	2	3	4	5	6	7	8	9	10
Trust(T)	0.50	0.48	0.52	0.59	0.70	0.79	0.87	0.74	0.56	0.50
Confidence Interval	Default	Less	Further Evaluation	Relatively	Further Evaluation	Further Evaluation	Further Evaluation	Unstable State	Unstable State	Default
	Reliability	Reliable		Reliable						Reliability
Trust Change (T^,^)	-	-0.02	0.04	0.07	0.11	0.09	0.08	-0.13	-0.18	-0.06
Time	11	12	13	14	15	16	17	18	19	20
Trust(T)	0.46	0.58	0.59	0.56	0.61	0.54	0.62	0.58	0.61	0.60
Confidence Interval	Less	Unstable State	Relatively	Relatively	Relatively	Relatively	Relatively	Relatively	Relatively	Relatively
	Reliable		Reliable	Reliable	Reliable	Reliable	Reliable	Reliable	Reliable	Reliable
Trust Change (T^,^)	-0.04	0.12	0.01	-0.03	0.05	-0.07	0.08	-0.04	0.03	-0.01
B. Trust Entities in Fig 6B										
Trust(T)	0.50	0.48	0.52	0.41	0.50	0.78	0.71	0.77	0.61	0.58
Confidence Interval	Default	Less	Further Evaluation	Unstable State	Unstable State	Unstable State	Further Evaluation	Further Evaluation	Further Evaluation	Relatively
	Reliability	Reliable								Reliable
Trust Change (T^,^)	-	-0.02	0.04	-0.11	0.09	0.28	-0.07	0.06	-0.16	-0.03
Time	11	12	13	14	15	16	17	18	19	20
Trust(T)	0.69	0.56	0.63	0.79	0.61	0.53	0.58	0.49	0.56	0.61
Confidence Interval	Further Evaluation	Further Evaluation	Relatively	Further Evaluation	Further Evaluation	Relatively	Relatively	Further Evaluation	Further Evaluation	Relatively
			Reliable			Reliable	Reliable			Reliable
Trust Change (T^,^)	0.11	-0.13	0.07	0.16	-0.18	-0.08	0.05	-0.09	0.07	0.05

**Table 9:** Statistics based on changes.

Trusted Entity	Frequency with a change greater than 0.1	Out of discrete frequency	Frequency of exceeding threshold and evaluation set	Total
a	4	3	1	8
b	5	5	8	18

In the above state diagram, if we take the most traditional trust state at a certain moment as the first benchmark for trust evaluation, then the state before the trust mutation of the entity is consistent with the current basic state of trust. After the sudden change in trust is lower than the default trust value, the trust status at this time is basically in line with the change in trust. When the subsequent trust value returns to the original trust state, the trust state at this point is certainly more credible, but it does not conform to the untrustworthy state experienced by this entity in the process of humility. At this time, due to the existence of the sudden change, the trust state does not completely conform to the actual situation. Therefore, only the instantaneous trust value is used to judge the trust state of the entity, which is inherently biased.

We feedback on the analogy of the complexity of evaluating trust based on weights and balance factors in trust analysis. One thing that is clear in the analogy is that the time complexity operation of the comparison method increases as the number increases. What is roughly clear is that when the amount of calculation of the constructed algorithm increases, the time complexity of the method increases with the increase of the number of algorithms and calculation definitions.

On the premise that we use the same evaluation index system, this part mainly analyzes the ratio of the time consumed to calculate the trust value and the evaluation system of the trust value. The figure shows that our method is superior to other methods in the following points: the time complexity based on the rate of change is relatively minimal, but with the addition of the weighting algorithm, the time complexity has a certain increase. The introduction of the weighting algorithm increases the time complexity of the classification system, and due to the existence of weights and it is difficult to change with the state of the entity, it may also produce uncertainty on the results of the trust value, and it is difficult to determine the accuracy and the real-time aspect guarantees the evaluation of trusted entities.

Although the method we propose here based on the rate of change and frequency is relatively low in complexity, we have a certain improvement in accuracy with a small amount of calculation. This shows that our method is effective and outperforms other methods in terms of cost performance.

## 5. Conclusions

When judging the trustworthiness of the terminal based on IoT, only using trust value to judge the reliability defects in the trust judgment lag and the trust value is not sensitive to short-term mutations. Therefore, this paper introduces the concept of rate of change and takes it as the trust value Reliability is determined based on attributes. By introducing the concept of rate of change, dynamic analysis of trust relationships and changes in trust is made, and a specific analysis of changes in entity trust values is given based on the synthesis of simulation and some public data. On the basis of the above analysis, a discrete interval evaluation set and a trust change frequency model are constructed respectively to judge the reliability of the trust state from the trust value, the rate of change, and the frequency of large changes to the trust state of the terminal entity. Compared with the traditional discrete and probabilistic trust analysis and determination mechanisms, introducing the rate of change as an attribute to the trust determination can have a more comprehensive reflection of the safety and reliability of trust and representatives.

However, in order to more effectively study the impact of changes in trust, and also limited by objective factors such as machine performance, this article only analyzes the security status of the terminal entity starting from the initial trust value from the unary variable of time. The future work is to change the research of trust change from one element to multiple, and analyze the influence of the change rate on trust from multiple factors such as time and interaction. At the same time, the work of this paper also hopes to be used in other fields based on trust analysis, which can actually promote this concept and get a more complete application.

## Supporting information

S1 File (DOCX)
