# Supplementary material for: IoT terminal security assessment system based on improved assessment method
Source: PLoS One. 2021 Sep 8;16(9):e0256881. doi: 10.1371/journal.pone.0256881 (PMC8425574; doi:10.1371/journal.pone.0256881)
Supplement: S1 File — (DOCX) [file pone.0256881.s001.docx]

This part of the data is the original of our previous experiment, which is attached to the article in the form of text schedule in this revision. Here, the data used for analysis shall be separated into volumes as required, and the table notes appearing in the text shall be reserved for reference and analysis in the future. Among them, Trust（T）represents the obtained data, and Trust Change($T^{,}$) represents the change amplitude data after calculation and analysis.

At the same time, it should be added that the above data represent a stage of trust implementation evaluation. In a variety of entities, the trust value measured in real time will also change due to various factors, but the method used can still be used to evaluate the change status.

Table 3 Stable Entity Trust Changes in Fig. 2a

| Time | 1 | 2 | 3 | 4 | 5 | 6 | 7 | 8 | 9 | 10 |
| --- | --- | --- | --- | --- | --- | --- | --- | --- | --- | --- |
| Trust(T) | 0.58 | 0.57 | 0.61 | 0.59 | 0.63 | 0.66 | 0.68 | 0.63 | 0.60 | 0.57 |
| Trust  Change  ($T^{,}$) | - | -0.01 | 0.04 | -0.02 | 0.04 | 0.03 | 0.02 | -0.05 | -0.03 | -0.03 |
| Time | 11 | 12 | 13 | 14 | 15 | 16 | 17 | 18 | 19 | 20 |
| Trust(T) | 0.56 | 0.54 | 0.56 | 0.60 | 0.62 | 0.63 | 0.60 | 0.59 | 0.58 | 0.56 |
| Trust  Change  ($T^{,}$) | -0.01 | 0.02 | 0.02 | 0.04 | 0.02 | 0.01 | -0.03 | -0.01 | -0.01 | -0.02 |

Table 4 Stable Entity Trust Changes in Fig. 3a

| Time | 1 | 2 | 3 | 4 | 5 | 6 | 7 | 8 | 9 | 10 |
| --- | --- | --- | --- | --- | --- | --- | --- | --- | --- | --- |
| Trust(T) | 0.52 | 0.54 | 0.51 | 0.53 | 0.55 | 0.56 | 0.54 | 0.53 | 0.58 | 0.56 |
| Trust  Change  ($T^{,}$) | - | 0.02 | -0.03 | 0.02 | 0.02 | 0.01 | -0.02 | -0.01 | 0.05 | -0.02 |
| Time | 11 | 12 | 13 | 14 | 15 | 16 | 17 | 18 | 19 | 20 |
| Trust(T) | 0.62 | 0.63 | 0.65 | 0.64 | 0.66 | 0.62 | 0.67 | 0.64 | 0.66 | 0.68 |
| Trust  Change  ($T^{,}$) | 0.06 | 0.01 | 0.02 | -0.01 | 0.02 | -0.04 | 0.05 | -0.03 | 0.02 | 0.02 |

Table 5a Trust Entities in Fig. 4a

| Time | 1 | 2 | 3 | 4 | 5 | 6 | 7 | 8 | 9 | 10 |
| --- | --- | --- | --- | --- | --- | --- | --- | --- | --- | --- |
| Trust(T) | 0.89 | 0.84 | 0.83 | 0.80 | 0.77 | 0.74 | 0.71 | 0.73 | 0.75 | 0.70 |
| Confidence  Interval | Highly  reliable | Highly  reliable | Highly  reliable | Highly  reliable | Highly  reliable | **Further Evaluation** | Relatively  Reliable | Relatively  Reliable | Relatively  Reliable | Relatively  Reliable |
| Trust  Change  ($T^{,}$) | - | -0.05 | -0.01 | -0.03 | -0.03 | -0.03 | -0.03 | 0.02 | 0.02 | -0.05 |
| Time | 11 | 12 | 13 | 14 | 15 | 16 | 17 | 18 | 19 | 20 |
| Trust(T) | 0.67 | 0.65 | 0.63 | 0.69 | 0.67 | 0.65 | 0.60 | 0.58 | 0.61 | 0.63 |
| Confidence  Interval | Relatively  Reliable | Relatively  Reliable | Relatively  Reliable | Relatively  Reliable | Relatively  Reliable | Relatively  Reliable | Relatively  Reliable | Relatively  Reliable | Relatively  Reliable | Relatively  Reliable |
| Trust  Change  ($T^{,}$) | -0.03 | -0.02 | -0.02 | 0.06 | -0.02 | -0.02 | -0.05 | -0.02 | 0.03 | 0.02 |

Table 5b Trust Entities in Fig. 4b

| Time | 1 | 2 | 3 | 4 | 5 | 6 | 7 | 8 | 9 | 10 |
| --- | --- | --- | --- | --- | --- | --- | --- | --- | --- | --- |
| Trust(T) | 0.80 | 0.76 | 0.75 | 0.74 | 0.78 | 0.61 | 0.55 | 0.70 | 0.54 | 0.52 |
| Confidence  Interval | Highly  reliable | Highly  reliable | Highly  reliable | **Further Evaluation** | **Further Evaluation** | **Unstable State** | Relatively  Reliable | **Unstable State** | **Unstable State** | Relatively  Reliable |
| Trust  Change  ($T^{,}$) | - | -0.04 | -0.01 | -0.01 | 0.04 | -0.17 | -0.06 | 0.15 | -0.16 | -0.02 |
| Time | 11 | 12 | 13 | 14 | 15 | 16 | 17 | 18 | 19 | 20 |
| Trust(T) | 0.67 | 0.81 | 0.74 | 0.72 | 0.61 | 0.75 | 0.72 | 0.71 | 0.69 | 0.72 |
| Confidence  Interval | **Unstable State** | **Unstable State** | **Further Evaluation** | Relatively  Reliable | **Unstable State** | **Unstable State** | **Further Evaluation** | Relatively  Reliable | Relatively  Reliable | Relatively  Reliable |
| Trust  Change  ($T^{,}$) | 0.15 | 0.14 | -0.07 | -0.02 | -0.11 | 0.14 | -0.03 | -0.01 | -0.02 | 0.03 |

Table 6 Default Trust Values in Fig. 5

| Time | 1 | 2 | 3 | 4 | 5 | 6 | 7 | 8 | 9 | 10 |
| --- | --- | --- | --- | --- | --- | --- | --- | --- | --- | --- |
| Trust(T) | 0.50 | 0.48 | 0.51 | 0.52 | 0.50 | 0.49 | 0.46 | 0.48 | 0.51 | 0.49 |
| Confidence  Interval | Default  Reliability | Less  Reliable | **Further Evaluation** | Relatively  Reliable | Relatively  Reliable | Less  Reliable | Less  Reliable | Less  Reliable | **Further Evaluation** | **Further Evaluation** |
| Trust  Change  ($T^{,}$) | - | -0.02 | 0.03 | 0.01 | -0.02 | -0.01 | -0.03 | 0.02 | 0.03 | -0.02 |
| Time | 11 | 12 | 13 | 14 | 15 | 16 | 17 | 18 | 19 | 20 |
| Trust(T) | 0.50 | 0.52 | 0.54 | 0.51 | 0.46 | 0.50 | 0.57 | 0.55 | 0.56 | 0.52 |
| Confidence  Interval | Further Evaluation | Relatively  Reliable | Relatively  Reliable | Relatively  Reliable | Further Evaluation | Further Evaluation | Relatively  Reliable | Relatively  Reliable | Relatively  Reliable | Relatively  Reliable |
| Trust  Change  ($T^{,}$) | 0.01 | 0.02 | 0.02 | -0.03 | -0.05 | 0.04 | 0.07 | -0.02 | 0.01 | -0.04 |

Table 7a Trust Entities in Fig. 6a

| Time | 1 | 2 | 3 | 4 | 5 | 6 | 7 | 8 | 9 | 10 |
| --- | --- | --- | --- | --- | --- | --- | --- | --- | --- | --- |
| Trust(T) | 0.50 | 0.48 | 0.52 | 0.59 | 0.70 | 0.79 | 0.87 | 0.74 | 0.56 | 0.50 |
| Confidence  Interval | Default  Reliability | Less  Reliable | Further Evaluation | Relatively  Reliable | Further Evaluation | Further Evaluation | Further Evaluation | Unstable State | Unstable State | Default  Reliability |
| Trust  Change  ($T^{,}$) | - | -0.02 | 0.04 | 0.07 | 0.11 | 0.09 | 0.08 | -0.13 | -0.18 | -0.06 |
| Time | 11 | 12 | 13 | 14 | 15 | 16 | 17 | 18 | 19 | 20 |
| Trust(T) | 0.46 | 0.58 | 0.59 | 0.56 | 0.61 | 0.54 | 0.62 | 0.58 | 0.61 | 0.60 |
| Confidence  Interval | Less  Reliable | Unstable State | Relatively  Reliable | Relatively  Reliable | Relatively  Reliable | Relatively  Reliable | Relatively  Reliable | Relatively  Reliable | Relatively  Reliable | Relatively  Reliable |
| Trust  Change  ($T^{,}$) | -0.04 | 0.12 | 0.01 | -0.03 | 0.05 | -0.07 | 0.08 | -0.04 | 0.03 | -0.01 |

Table 7b Trust Entities in Fig. 6b

| Time | 1 | 2 | 3 | 4 | 5 | 6 | 7 | 8 | 9 | 10 |
| --- | --- | --- | --- | --- | --- | --- | --- | --- | --- | --- |
| Trust(T) | 0.50 | 0.48 | 0.52 | 0.41 | 0.50 | 0.78 | 0.71 | 0.77 | 0.61 | 0.58 |
| Confidence  Interval | Default  Reliability | Less  Reliable | Further Evaluation | Unstable State | Unstable State | Unstable State | Further Evaluation | Further Evaluation | Further Evaluation | Relatively  Reliable |
| Trust  Change  ($T^{,}$) | - | -0.02 | 0.04 | -0.11 | 0.09 | 0.28 | -0.07 | 0.06 | -0.16 | -0.03 |
| Time | 11 | 12 | 13 | 14 | 15 | 16 | 17 | 18 | 19 | 20 |
| Trust(T) | 0.69 | 0.56 | 0.63 | 0.79 | 0.61 | 0.53 | 0.58 | 0.49 | 0.56 | 0.61 |
| Confidence  Interval | Further Evaluation | Further Evaluation | Relatively  Reliable | Further Evaluation | Further Evaluation | Relatively  Reliable | Relatively  Reliable | Further Evaluation | Further Evaluation | Relatively  Reliable |
| Trust  Change  ($T^{,}$) | 0.11 | -0.13 | 0.07 | 0.16 | -0.18 | -0.08 | 0.05 | -0.09 | 0.07 | 0.05 |
